# Impact of Climate Change on Cellulitis: A Literature Review

**DOI:** 10.7759/cureus.64958

**Published:** 2024-07-19

**Authors:** Ahmad A Rathor, Michelle Lin, Rodger D MacArthur

**Affiliations:** 1 Office of Academic Affairs, Augusta University Medical College of Georgia, Augusta, USA; 2 Division of Infectious Diseases, Augusta University Medical College of Georgia, Augusta, USA

**Keywords:** global temperature, gram-positive bacteria, sea bacteria, ssti, bacterial skin infection, weather, global warming, climate change, cellulitis

## Abstract

Climate change is a phenomenon that has had, and will continue to have, wide-ranging effects on the world in both the near and distant future. With regards to human health, research has demonstrated the impact of climate change on heat-related illness, mental health, and vector-borne infectious diseases. Through a review of the literature, this paper aims to elucidate both current and future consequences of climate change on cellulitis, a type of skin infection that is associated with significant morbidity, mortality, and cost. Factors such as elevated temperature, pollution, rising sea levels, and the increased frequency of natural disasters pose an alarming risk for the increased proliferation of infections such as cellulitis. Lastly, in light of these trends, this paper will address potential strategies individuals can implement to reduce the effects of climate change on cellulitis.

## Introduction and background

Cellulitis is among the most common infections that afflict humans and is defined as a bacterial infection of the deep dermis and the adjacent subcutaneous tissue [1]. Cellulitis presents as a warm, erythematous region of the skin that is often associated with swelling and tenderness [1]. It is often grouped under the larger category of skin and soft tissue infections (SSTIs), a classification of infections that also includes abscesses, erysipelas, folliculitis, and impetigo.

Research has shown that cellulitis develops due to injury of the skin, an essential component of innate immunity that serves as a barrier to exogenous pathogens [1]. Once the skin is broken, the pathogen migrates to the dermis and subcutaneous tissue, often producing peptides that result in an immune response involving neutrophil recruitment [1]. The vast majority of cases of cellulitis occur due to bacteria; however, the specific bacterium is often unknown as the bulk of cellulitis presentations are difficult to culture [1]. In the minority of cellulitis cases in which an organism was identified, beta-hemolytic streptococci and methicillin-sensitive *Staphylococcus aureus* were found as the most common culprits [1]. Given this, cellulitis is most commonly treated with antibiotics that are effective against streptococcal species, although certain patient risk factors may require the use of a different antimicrobial with broader pathogen coverage [1].

Cellulitis has several risk factors, including old age, obesity, venous insufficiency, and diabetes mellitus [2]. Furthermore, specific populations including children, athletes, men who have sex with men, intravenous drug users, military recruits, and residents of long-term care facilities are at risk for infection with methicillin-resistant *Staphylococcus aureus* (MRSA), a strain of bacteria that is resistant to multiple antibiotics and provides clinicians with a treatment challenge [1].

In the United States, current estimates suggest that there are more than 14 million cases of cellulitis annually [1]. Cellulitis most commonly occurs in middle-aged and older adults, with the incidence increasing as age increases [3]. There has been no significant association with patient sex [3]. Although many cellulitis cases are diagnosed in urgent and primary care clinics and treated on an outpatient basis, some presentations require a higher level of care. In the United States, estimates suggest that cellulitis results in over 650,000 hospital admissions every year [1]. Associated with the high incidence of the condition is an incredible financial burden on the American healthcare system. In fact, a 2016 study estimates that cellulitis accounts for $3.7 billion in ambulatory costs every year [1]. With regards to the inpatient setting, adult hospitalization with a primary diagnosis of cellulitis resulted in almost $4 billion in costs [4].

## Review

Methodology

The primary methodology for the review consisted of searching the PubMed and Google Scholar databases for relevant manuscripts. Specific search terms used included "cellulitis AND temperature," "cellulitis AND weather," "cellulitis AND hurricane," "cellulitis AND typhoon," "cellulitis AND pollution," and "cellulitis AND ozone." Articles were screened for their relevance to discussing the effects of temperature, tropical cyclones, rising sea levels, pollution, and wildfires. To illustrate and quantify the past, present, and future of climate change, internet search engines were used to obtain background information from reputable national and international organizations. As specific articles were reviewed, additional ones were added to our corpus of sources by reviewing relevant references and performing more targeted searches on PubMed and Google Scholar. Only articles published in English were included. Furthermore, there was no restriction placed on study design. Lastly, there was no restriction placed on the date of publication.

Elevated temperature

One of climate change’s most notorious effects has been its impact on global temperature. Since 1850, the surface temperature of Earth has increased by approximately 2 degrees Fahrenheit [5]. Moreover, the rate of increase since 1982 is more than three times as fast [5]. As a result, the warmest year in recorded human history was 2023, with the other nine warmest years all occurring in the past decade [5]. In fact, 2023 was 2.12 degrees Fahrenheit higher than the 20th century average of 57.0 degrees Fahrenheit [5]. Climate scientists have declared that the severity of future warming is linked to the volume of greenhouse gases released [5]. If the volume of greenhouse gas emissions continues its rapid ascent, experts predict that by the year 2100, global temperature will be 5-10.2 degrees Fahrenheit warmer than the 1901-1960 average [5].

Seasonality has been implicated in the incidence of cellulitis throughout the literature. Using data from United States inpatient records and cross-referencing with data from the National Climatic Data Center, Peterson et al. revealed that there was an elevated incidence of cellulitis in the summer months, with the peak incidence in July being 35% greater than the nadir incidence in February [4,6]. Seasonality is associated with several factors such as the direction of the Earth’s axis relative to the sun, temperature, humidity, and precipitation. Higher temperatures in the summer months were the major driver behind the seasonal association of cellulitis [4]. In fact, over 70% of the observed seasonality of cellulitis could be explained by considering the average monthly temperature [4]. The study also demonstrated a strong dose-dependent relationship between average monthly temperatures and the risk of cellulitis [4]. Specifically, the study elucidated that for every 5 degree Fahrenheit rise in temperature, the odds of a patient with cellulitis being admitted to the hospital increased by approximately 3.55% [4]. The association between elevated temperatures and cellulitis was present even after controlling for age, sex, length of stay, geographic region, latitude, and longitude [4]. Moreover, the means by which patients received healthcare services did not alter the association between increased temperatures and cellulitis [4]. Furthermore, a study on surgical site infections, including cellulitis, demonstrated an association with higher temperatures [7].

As mentioned previously, cellulitis most commonly occurs in middle-aged and older adults, with the incidence increasing as age increases [3]. Despite the lower incidence of cellulitis in younger individuals, the association between increased temperature and cellulitis has also been demonstrated in pediatric populations. A study involving patients aged 0-19 years in Arizona revealed a seasonal pattern of SSTIs, discovering a peak of cases in early September [8]. Furthermore, scientists found a significant correlation between the temporal variation in SSTI incidence and the average temperature in this pediatric population [8]. 

The association between seasonality and cellulitis has been demonstrated beyond the United States, in several regions of the world with their own distinct weather patterns. In the East Anglia region of the United Kingdom, Haydock et al. reviewed admission data over a three-year period from the mid-2000s and discovered that admissions for non-necrotizing cellulitis are markedly increased in the late spring and summer months with a peak in May [9]. In Western Australia, the total number of primary cases of lower leg cellulitis was higher in both the summer and autumn [10]. In Saudi Arabia, a retrospective review of Hajj pilgrim patients of 42 different nationalities between 2004 and 2012 showed that cellulitis accounted for approximately one-quarter of general surgical admissions with proportional increases from 2004 to 2012 [11]. Additionally, there was a statistically significant correlation with the rise in seasonal temperatures [11]. Moreover, a retrospective study of almost 200,000 patients diagnosed with cellulitis in Taiwan revealed that the incidence rate was strongly correlated with both temperature and total sunshine hours [12]. When trying to quantify the strength of temperature, the researchers found that the incidence rate of cellulitis increased by 3.47/100,000 cases for every 1 degree Celsius elevation in environmental temperature [12].

In the United States, the overall number of cellulitis cases has been increasing. Spanning a time period of 1998-2013, researchers have shown that hospitalizations for cellulitis in the United States have doubled [6]. Furthermore, their findings revealed that the increasing incidence of cellulitis is highly seasonal, with hospitalizations reaching a climax during the summer months [6]. Given the dose-dependent relationship between temperature and cellulitis, the significantly increased incidence of cellulitis in 2013 compared to 1998 may be partially explained by the increase in average temperature that has occurred during this timeframe [4]. 

The exact cause of the link between elevated temperature and cellulitis is a target for future research. Most cases of SSTIs due to *Staphylococcal aureus* arise from bacteria that have already colonized the patients’ skin or mucosa [13]. Additionally, direct contact is the primary mode of *Staphylococcal aureus* transmission from one person to another [13]. As a result, as Leekha et al. note, weather changes that increase host colonization, susceptibility to infection, and person-to-person transmission could serve as an explanation for this potential link [13]. Studies have demonstrated that bacterial populations on the back, axillae, and feet are significantly greater for people in high-temperature and high-humidity environments compared with moderate-temperature, low-humidity environments [13,14]. Moreover, because insect bites are associated with skin injury, they are implicated in the pathogenesis of many cases of cellulitis [15]. A study in England and Wales discovered that the number of insect bites peaked with temperature and that there was an association between insect bites and impetigo, another type of SSTI [16].

Tropical cyclones and rising sea levels

Tropical cyclones are rapidly rotating storms that include the subcategories of hurricanes and typhoons [17]. Collectively, these tropical storms are the second-most dangerous natural hazard causing almost 800,000 deaths within the last half century [17]. Increased ocean surface temperatures due to climate change have been implicated in the formation of these tropical cyclones [18]. Moreover, in addition to increasing the incidence, climate change is also associated with increased severity of hurricanes [17]. Directly, tropical cyclones can cause property damage, injury, and loss of life due to heavy rains and strong winds [17]. 

In addition, the powerful winds of tropical cyclones cause tsunami-like storm surges [17]. The large degree of ocean water propelled towards the shore can result in severe flooding [17]. Apart from the effect of tropical cyclones, climate change is implicated in the rise of sea levels [19]. This increase is attributed to the expansion of ocean water due to warming as well as the melting of glaciers and ice [19]. Due to these changes, scientists estimate that high-tide flooding is now 300% to 900% more common compared to half a century ago [19]. Compared to 1880 figures, the global average sea level has increased by 8-9 inches [19].

The incidence of infections with *Vibrio*, a cause of cellulitis that is common in ocean water, has been increasing both in the United States and around the globe, likely due to ocean warming [20]. Traditionally, the majority of *Vibrio* cases occur in the summer months [20]. As the ocean continues to warm, the rising incidence of *Vibrio* infections is postulated to persist [20]. *Shewanella*, another genus of marine bacteria, has been implicated in several cases of cellulitis where patients have had exposure to marine water [21-24]. Other bacteria that are common causes of cellulitis after marine water exposure include *Aeromonas hydrophila* and *Chromobacterium violaceum* [25]. These atypical causes of cellulitis provide both a diagnostic and treatment challenge for clinicians [24]. *Shewanella algae*, for example, has demonstrated resistance against cephalosporins and penicillins, two classes of antibiotics that are considered first-line treatment for SSTIs [26]. In addition, resistance to quinolones and carbapenems has been shown in the literature [26,27]. Moreover, *Vibrio vulnificus* is associated with severe cases of infections that can cause upwards of 20% of patients to die, with many patients requiring ICU-level care and/or amputations [28].

Heavy rainfall is an integral component of the danger posed by tropical cyclones. Precipitation has been demonstrated as a risk factor for cellulitis across multiple studies. In Taiwan, researchers elucidated that both total rainfall and the total number of rainy days were strongly associated with the incidence of cellulitis [12]. In addition, increased relative humidity was strongly associated with a higher incidence of cellulitis [12]. Similarly, in a study analyzing a 2016-2018 epidemic of necrotic skin infections in São Tomé and Príncipe, researchers discovered that increased relative humidity was associated with increased monthly cases [29]. The researchers also found that higher amounts of precipitation in the previous month were associated with a higher number of cases in the subsequent month.

Moreover, the human cost of tropical cyclones is immense and includes injuries that can predispose patients to cellulitis. Following Typhoon Haitang that ravaged Taiwan in 2005, researchers noted an increased incidence of cellulitis in the two-week period following the typhoon compared to the two-week period prior to the typhoon [30]. In addition, investigators found that over one-fourth of patients with cellulitis after the typhoon had their affected extremities submerged in flood water, suggesting a possible mechanism for the start of the infection [30]. Similarly, *Vibrio vulnificus* was a common source of infection after Hurricane Katrina, a powerful Category 5 hurricane that devastated regions of the southern United States in 2005 [31]. Despite the lower incidence of cellulitis in younger populations, the association between tropical cyclones and cellulitis has also been discovered in pediatric populations. In 1992, following Hurricane Andrew, another powerful Category 5 hurricane, doctors at a Miami children’s hospital noted a higher number of patients being diagnosed with cellulitis and open wounds [32]. Following Hurricane Sandy in 2012, trauma surgeons observed an increased incidence of cut injuries following the hurricane [33]. The surgeons established that these injuries were caused by both the hurricane itself as well as from the recovery process [33]. Similarly, Hurricane Maria in 2017 was associated with an increased incidence of fall-related injuries [34]. Trauma to the skin, such as from a fall-related injury, can result in cellulitis due to the introduction of microorganisms [35].

Pollution

Pollutants have played a tremendous role in the climate change crisis. Air pollutants such as black carbon and methane are termed short-lived climate pollutants and have a significant effect on global warming [36]. Ozone, another potent air pollutant, is a derivative of methane [36]. Ozone, commonly referred to by its chemical formula of O_3_, is a gas composed of three atoms of oxygen [37]. Although naturally found in the upper atmosphere, ozone at the ground level is harmful and serves as one of the critical constituents of smog [37]. Ozone is synthesized via chemical reactions involving nitrogen oxides, volatile organic compounds, and heat [37]. Sunlight is another reagent involved in the chemical reactions that produce ozone [37]. In particular, the amount of ambient ground level of ozone is believed to increase after exposure to ultraviolet-B light, a type of ultraviolet light emitted by the sun [38]. The sources of nitrogen oxides and volatile organic compounds that result in the generation of ozone include modes of physical transportation, such as cars, as well as industrial facilities such as chemical refineries [37]. Given these risk factors, ozone levels are higher in warmer, urban environments [37]. However, one of ozone’s most virulent features is its ability to be carried long distances via wind, allowing it to penetrate non-urban communities as well [37].

Particulate matter with a median aerodynamic diameter of less than 2.5 μm and nitrogen dioxide, both harmful pollutants, have been shown to have immediate effects on the incidence of cellulitis [39]. Moreover, a positive correlation has been established between exposure to ozone and the incidence of cellulitis due to *Streptococcus* species [40]. Additionally, an increased quantity of ground-level ozone was associated with an increased number of emergency room visits for cellulitis [41]. The effect of ozone on cellulitis was also mediated by age, with infants and children presenting more often for cellulitis [41]. Although the majority of research suggests that the risk of cellulitis increases as age increases, the study surprisingly demonstrated that there was an elevated risk of cellulitis associated with ozone for adults 65 years of age and younger [3,41].

The exact mechanism of ozone’s impact on skin is a topic of continuing research. In chickens, ozone has been linked to structural damage of the skin, causing the skin to have increased permeability to bacteria [42]. Also, studies have demonstrated that ozone is a compound that activates the oxidative stress response in rodent skin [43]. In particular, ozone upregulates heat shock protein 27 and nitric oxide synthase [43]. Moreover, in vivo exposure to ozone depletes vitamins C and E and induces the peroxidation of lipids in the epidermis of rodent skin [44]. These various biochemical changes damage nucleic acids, proteins, and cellular membranes, resulting in injury of the skin cells [45,46]. Furthermore, ultraviolet-B light, a reagent in the synthesis of ozone, has been demonstrated to cause prominent immunosuppression via inhibition of T cells [47]. As a result, the ultraviolet-B radiation that helps produce ozone, as well as ozone itself, poses a synergistic threat that increases the risk of skin conditions such as cellulitis [45]. Pollution has also been shown to increase the risk of skin conditions like acne, atopic dermatitis, psoriasis, and skin cancer, suggesting that there may be other cellular changes associated with pollution that may also increase the risk of cellulitis [45].

Wildfires

Wildfires have been classified as an indicator of climate change according to the United States Environmental Protection Agency [48]. In the past few decades, temperature and rainfall changes in the western region of the United States have contributed to a warmer, drier climate that has served as a catalyst for wildfires [48]. These changes have also resulted in the peak of the United States wildfire season shifting from August to July [48]. Furthermore, the climate crisis is threatening to increase the frequency and strength of wildfires in the future [48]. Although wildfires often begin in sparsely populated areas, they have the potential to spread and cause devastation to residential communities across the world. Moreover, among the different categories of burns, wildfire-associated burns are especially devastating. A 2021 study concluded that wildfire burn patients are potentially at an elevated risk of wound infection, mortality, length of stay, and readmission [49].

In a study evaluating patients with lower extremity burns over a six-year period, researchers observed that cellulitis was the second-most common complication observed [50]. Due to skin injury, burns are a tremendous risk factor for the initial colonization of gram-positive bacteria that tend to be sensitive to antibiotic treatment [51,52]. However, as time progresses, these wounds become more susceptible to gram-negative organisms that are often resistant to multiple antimicrobials [51,52]. In fact, multidrug-resistant pathogens are frequent causes of nosocomial infections such as cellulitis and are one of the most common reasons for mortality in patients with burns [52]. If the frequency and power of wildfires continue to increase, the risk of burn-related injuries and sequelae such as cellulitis are poised to increase as well.

Mitigation strategies

Climate change is an incredibly complex crisis that necessitates collaboration between a plethora of different stakeholders, including local, state, and national governments, international diplomatic organizations such as the United Nations, national and international companies, religious institutions, nonprofit organizations, and institutes of education. Despite the overwhelming difficulty associated with tackling this crisis, there are several strategies that individuals can employ to protect their skin against cellulitis within the context of the changing climate. 

One important strategy is to increase the coverage of skin when outdoors. As mentioned previously, skin injury is implicated in the development of cellulitis. In a study analyzing collegiate football players, Bowers et al. noted that increased perspiration due to the warm weather and weakening of the skin via abrasions and "turf burn" were all factors that contributed to the higher incidence of MRSA cellulitis [53]. Although not formally studied, they hypothesized that players wearing more skin-covering clothing due to the colder weather reduced the risk of cutaneous MRSA infection by preventing skin injury [53]. Researchers have hypothesized that skin injuries caused by outdoor activities such as swimming can also be a key reason for the increased incidence of cellulitis in the summer months [12]. Furthermore, since insect bites are implicated in the development of many cases of cellulitis, wearing clothing will invariably reduce the amount of exposed skin available for insects [15,54]. Also, wearing protective gear while in close proximity to ocean water will likely reduce exposure to marine bacteria like *Shewanella algae* and *Vibrio vulnificus* that can cause cellulitis.

While clothing can protect against macroscopic injury to the skin like cuts or scrapes, it may serve a protective role at the microscopic level as well. The association between increased amounts of ozone and cellulitis was more pronounced in skin areas that have greater exposure to the air, such as the arms, face, and neck, when compared to areas that tend to be more covered, such as the trunk and legs [41]. In addition, human-to-human transmission has been implicated in the development of cellulitis. In the aforementioned study on collegiate football players, cutaneous MRSA infections only began to occur after players were exposed to increasing levels of physical contact with their teammates [53]. Skin-to-skin contact more generally, and contact with the sweat of other players specifically, may increase the transmission of MRSA and the risk of subsequent skin infections [53]. Similarly, in Taiwan, researchers theorized that the increased incidence of cellulitis in the summer corresponded with the start of the school year, a time of the year when overcrowding can ease the spread of pathogens [12].

Although clothing can serve a protective role against cellulitis by preventing skin injury and reducing pathogen transmission, it can serve as a vehicle for the spread of bacteria when shared amongst groups of people. A report from the Centers for Disease Control and Prevention (CDC) concluded that bacteria on shared clothing and equipment was the most likely culprit of a cluster of MRSA skin infections in players of a Colorado fencing club, a sport with little skin-to-skin contact [55]. Recommendations from the CDC for the prevention of staphylococcal skin infections among sports players include showering and washing with soap after physical activity, limiting the sharing of towels and clothing, and regular assessment of the skin for lesions [55]. If the incidence of cellulitis continues to increase and climate change continues to increase the risk of cellulitis, these measures may also prove useful to individuals not engaged in sports.

Lastly, insect bites have been identified as a risk factor for cellulitis and other types of SSTIs by causing skin injury that results in bacterial infiltration [15,16]. Research has demonstrated a strong relationship between temperature and the number of insect bites, possibly due to increased insect activity and/or people spending more time outdoors [16]. Mosquitoes, a common cause of bites for people partaking in outdoor activities, have been greatly affected by climate change. Higher temperatures that are associated with the changing climate have been shown to promote mosquito growth and increase biting rates [56]. The CDC has recommended several strategies to prevent individuals from getting bug bites [54]. Insect repellants and spraying clothing with insecticides like permethrin can stave off mosquitoes and sandflies [54]. Furthermore, the CDC recommends utilizing door screens, mosquito nets, and covering strollers with netting [54]. If the changing climate continues to result in the proliferation of biting insects, these strategies will prove to be increasingly beneficial.

Limitations and future directions

One of the major limitations of our literature review is the lack of evidence establishing the underlying mechanisms behind several of the associations that were highlighted, such as the association between rainfall and cellulitis. In cases where there is an underlying mechanism that has been studied, such as with ozone, the data originates from animal studies instead of human studies. These unknowns regarding the pathophysiology are topics for future research.

Moreover, our literature review did not elucidate any evidence directly comparing the quantitative effect of the various climate-related factors that contribute to cellulitis. For instance, in the setting of increased temperature, do insect bites play a greater role in the pathogenesis of cellulitis than human-to-human contact, or vice versa? Furthermore, more research is needed to explore the impact of the mitigation techniques that were discussed. For example, in summer months, which are associated with higher temperatures, do individual patients who use insect repellent have a lower risk of cellulitis? Exploring the strength of these strategies on the individual level will help public health agencies prioritize those that are most effective for implementation on a broader scale.

Additionally, as Peterson et al. note, specific medical risk factors for cellulitis, such as lower extremity swelling, have associations with warmer weather [4]. The complex interplay between a patient’s medical history, climate change, and cellulitis is yet to be investigated. Also, although our review featured research from diverse parts of the world, there are many countries with unique climatic conditions that have yet to be studied. Lastly, we utilized articles that were only in English, which limited the scope of our review.

## Conclusions

The relationship between climate change and cellulitis is complex and involves multiple factors. Across multiple regions of the world, a relationship between increased temperature and the risk of cellulitis has been established. Natural disasters such as tropical cyclones and wildfires, which are projected to increase in frequency in the coming decades, can cause physical injuries that predispose patients to cellulitis. Moreover, the rising sea level increases the exposure to marine bacteria that can cause presentations of cellulitis that are difficult to treat. Furthermore, pollution causes microscopic injuries of the skin, permitting bacteria to enter the body and proliferate with ease. Tackling the climate crisis is an arduous endeavor that will require cooperation between the world’s largest and most influential institutions. On the individual level, strategies to prevent cellulitis include reducing exposed skin, limiting the sharing of clothing, and preventing insect bites. Moving forward, additional research exploring public health measures to protect skin from infection is needed in light of the multitude of dangers posed by the changing climate.
